# A multiobjective approach for identifying protein complexes and studying their association in multiple disorders

**DOI:** 10.1186/s13015-015-0056-2

**Published:** 2015-08-09

**Authors:** Sanghamitra Bandyopadhyay, Sumanta Ray, Anirban Mukhopadhyay, Ujjwal Maulik

**Affiliations:** Machine Intelligence Unit, Indian Statistical Institute, Kolkata, 700108 West Bengal India; Department of Computer Science and Engineering, Aliah University, Kolkata, 700156 West Bengal India; Department of Computer Science and Engineering, University of Kalyani, Kalyani, 741235 West Bengal India; Department of Computer Science and Engineering, Jadavpur University, Kolkata, 700032 West Bengal India

**Keywords:** Protein–protein interactions, Protein complex, Multiobjective optimization, Gene Ontology, Disorders

## Abstract

**Background:**

Detecting protein complexes within protein–protein interaction (PPI) networks is a major step toward the analysis of biological processes and pathways. Identification and characterization of protein complexes in PPI network is an ongoing challenge. Several high-throughput experimental techniques provide substantial number of PPIs which are widely utilized for compiling the PPI network of a species.

**Results:**

Here we focus on detecting human protein complexes by developing a multiobjective framework. For this large human PPI network is partitioned into modules which serves as protein complex. For building the objective functions we have utilized topological properties of PPI network and biological properties based on Gene Ontology semantic similarity. The proposed method is compared with that of some state-of-the-art algorithms in the context of different performance metrics. For the purpose of biological validation of our predicted complexes we have also employed a Gene Ontology and pathway based analysis here. Additionally, we have performed an analysis to associate resulting protein complexes with 22 key disease classes. Two bipartite networks are created to clearly visualize the association of identified protein complexes with the disorder classes.

**Conclusions:**

Here, we present the task of identifying protein complexes as a multiobjective optimization problem. Identified protein complexes are found to be associated with several disorders classes like ‘Cancer’, ‘Endocrine’ and ‘Multiple’. This analysis uncovers some new relationships between disorders and predicted complexes that may take a potential role in the prediction of multi target drugs.

**Electronic supplementary material:**

The online version of this article (doi:10.1186/s13015-015-0056-2) contains supplementary material, which is available to authorized users.

## Background

Recent advancement in biotechnology produces lots of information about protein–protein interactions. Those information act as a potential source to construct the protein–protein interaction network (PPIN) for a single species. Protein complexes are generally described as molecular aggregation of a set of proteins connected by multiple protein–protein interactions. Protein complexes play different functions in the cell. It can serve as cellular machines, rigid structures, and posttranslational modification systems. In general cellular functions and biochemical events in cell are coordinately performed by a groups of proteins interacting with each other in protein complexes. Identifying such protein complexes is important for understanding the structure and functions of these biochemical events. Moreover, changing of interaction pattern of proteins is the consequence of many diseases. Identifying such interactions through protein complexes predominantly lead to applications in disease diagnosis.

In usual representation, protein complexes take the form of a dense clusters of proteins connected through multiple interactions. Different computational methods for finding dense regions in the PPI network are available in literature. Several techniques based on graph clustering, finding dense regions, or clique finding have been proposed to discover protein complexes in PPI networks [1–4]. In [5] Molecular Complex Detection (MCODE) has been proposed to detect protein complexes in PPI network. MCODE generally emphasizes on the local neighborhood density of nodes and puts weight to all the nodes corresponding to the local density. Starting from the top weighted node it iteratively adds vertices which have weights above a certain threshold.

In [6] a clustering with overlapping neighborhood expansion (ClusterONE) is proposed for detecting overlapping protein complexes from protein–protein interaction data. ClusterONE generally follows a greedy procedure to update the partially constructed groups of vertices based on high cohesiveness among the vertices. The growth process is repeated from different seeds to form multiple, possibly overlapping groups. In the second step, overlapping groups are merged based on overlap scores.

In [7] an algorithm called Affinity Propagation is proposed which is basically an unsupervised algorithm and thus the number of clusters is automatically calculated. The data points are grouped based on the similarity between each pair of data points. Initially all data points are considered as potential “exemplars”. The main algorithm is concentrated on finding sub-paths, which allow easy message exchanges between nodes. In subsequent steps, the exchange of message is continued to happen between the nodes until a set of exemplars and corresponding clusters with high quality come out.

In [8] a multiobjective framework is proposed for detecting protein complexes in yeast PPI network. Here two different types of objectives are utilized for searching over the whole PPI network to predict modules which serve as protein complexes. Density of the module and Gene Ontology based semantic similarity measures are taken into account for building the objectives.

All these methods primarily focus on the detection of protein complexes in PPI network of model organism yeast (*S. cerevisiae*). Although, there have been several studies on determining and analyzing protein complexes in a single organism, computational analysis of human protein complexes is not studied in extensive manner. Some studies have analyzed human protein complexes based on a particular disease association [9–11]. These studies are focused on finding protein complexes associated with some specific disease. Here, we have proposed a multiobjective evolutionary technique for detecting protein complexes in human PPI network and studied their involvement in different disease classes.

In general, it has been observed that the proteins within a complex are functionally similar and carry out common biological activities. For measuring the functional similarity between two proteins we have computed semantic similarity between Gene Ontology terms associated with those proteins. In linguistic, to measure the similarity between two concepts, semantic similarity is used. This can be extended to measure the similarity between GO-terms in the GO database [12]. Here, we have utilized the Relevance [13] semantic similarity measure for obtaining semantic similarity between two proteins. This serves one of the objective function of our multiobjective framework. Besides the semantic similarity measure topological properties of PPI network are also used here for building the objective functions. Non dominated sorting genetic algorithm II (NSGA-II) [14, 15], a popular multiobjective Genetic Algorithm (GA) [16] has been utilized as the underlying optimization tool. The results are collected by applying the proposed algorithm in the protein–protein interaction (PPI) data downloaded from the Human Protein Reference Database (HPRD) [17]. The performance of our method is compared with that of some other existing methods such as MCODE [5], clusterONE [6], Affinity propagation [7], Core attachment method [18], COACH [19], RNSC [20], MCL-Caw [21], and PEWCC [22].

Here, we have reported the associations among predicted complexes with similar type of diseases/disorders. Identifying the associations between human protein complexes and multiple disorders is essential for understanding disease mechanism and is also important to assist drug developers for the development of new diagnostics and therapeutics. In Goh et al. [23], a bipartite network is formed that shows disorder-gene association which lead to the concept of ‘diseaseome’. In this network one set of nodes represent all known genetic disorders, and the other set corresponds to all known disease genes in the human genome. A disorder and a gene is connected by a link if the mutations in that gene is incriminated by that disorder. They found that genes associated with similar diseases have an increased tendency to interact with one another, and tend to exhibit high connectivity with each other forming a dense cluster. So it is necessary to discover the association of our predicted protein complexes with those genes causing similar diseases. For this purpose we have analyzed the predicted complexes and associated them with genes causing similar disease. We have searched the involvement of proteins within predicted complexes in 22 type of primary disease/disorder classes and found most of them are associated with ‘Cancer’ disease class. We have also formed two bipartite networks between all predicted protein complexes and disease/disorder classes. These networks show the involvement of protein complexes within disorders/disease classes. This may uncover interesting association or relationship between diseases and protein complexes. This can contribute significant effort to develop new strategies in Polypharmacological drug discovery which focus on multi-target drugs.

## Methods

In this section we describe the proposed multiobjective method for detecting protein complexes in human PPI network. Non-dominated Sorting GA (NSGA-II) [14] is employed as an underlying multiobjective framework.

### Chromosome representation

Here a protein complex (or a subgraph of human PPI graph) is encoded as a chromosome. It is represented as $p_1,p_2 \ldots p_n$ where $p_i$ is the ith protein in whole human protein set. Thus a chromosome represents a protein complex containing the nodes $p_1,p_2, \ldots p_n$, and the edges among them represent interactions.

### Population initialization

For starting from a reasonable position we construct the initial population as a set of modules with high density. For this purpose we randomly chose some substructure consisting of all 1s from adjacency matrix. To find out all 1s substructures we apply a biclustering technique [24] and randomly pick up some of the biclusters consisting of all 1s. Here, union of rows and columns of each bicluster is treated as a chromosome, which comprise the initial population.

### Representation of objective functions

Here two categories of objective functions are built, one is based on the topological characteristics of the network and the other captures gene ontological similarity of proteins. To define the two objective functions belonging to the first one we have incorporated some graph-based properties of the PPIN. For the other category, we employed GO based semantic similarity measure.

### Objective functions related to the topological properties

We have defined two objective functions in this category. One is based on the density of protein complexes, and other is based on closeness centralities. The density of a graph is defined as ratio of the number of edges present in a graph to the possible number of edges in a complete graph of same size. Protein complexes generally represent high dense area in the PPIN. Large number of interactions (or edges) among proteins (or nodes) in the complex is the possible reason behind that. Thus, using density as an objective function and maximizing it for individual subgraphs will yield much denser complexes.

For choosing the next objective we calculate the contribution of a node as follows: $Contr(n_i)=\frac{\mid N_{n_i} \mid }{degree(n_i)},$ where $N_{n_i}$ represents the set of nodes directly connected with node $n_i$ in a protein cluster *C*. Now the contribution of a protein cluster can be calculated as the summation of these values which can be formulated as:

1 $$\begin{aligned} \sum _{n_i \epsilon C} Contr(n_i). \end{aligned}$$

Maximizing this will produce clusters having small number of outward interaction partners for a node, thus producing compact as well as separated clusters.

Closeness centrality of a vertex in a graph is defined as the reciprocal of average shortest-path distance to other vertices. It can be considered as the efficiency of a node (individual) in spreading information to others in the network. Higher value of it indicates that most of the nodes are closer to that node. Here we maximize the objective function:

2 $$\begin{aligned} \sum _{n_i \epsilon C} CC(n_i), \end{aligned}$$

where *C* represents a protein cluster and $CC(n_i)$ is the closeness centrality of the node $n_i$. Maximizing this ensures that the resulting clusters have nodes which are more central in the whole protein interaction graph and are likely to form a protein complex.

### Objective function related to Gene Ontology

Proteins within the protein complexes are functionally similar to each other. This suggests that these proteins have high semantic similarity among themselves. This is measured by computing the semantic similarity between GO-terms they are annotated with. As proteins are annotated with multiple GO terms, so, the similarities are calculated by averaging the similarities of the GO term cross pairs which are associated with them [25]. We have calculated the similarities among all pairs of proteins in the PPI network and given these as weights of edges in semantic similarity network. The average similarity of all pairs of proteins corresponding to the edges of a chromosome is treated as fitness value of it. For example, the fitness of a chromosome or a subgraph is calculated by summing up all the weights of edges and averaging these values. This can be written as:

3 $$\begin{aligned} sim(s)=\frac{\sum _{i=1,j=1,i \ne j}^p w(n_i,n_j)}{p}, \end{aligned}$$

where *s* is the chromosome consisting of nodes $n_1,n_2, \ldots n_p$, $(n_i,n_j) \epsilon E$, where *E* is the set of edges and *w* is a weight function defined as $w: E \rightarrow [0,1]$.

### Mutation procedure

The usual genetic operators are selection, crossover, and mutation. Here crossover operation is not performed as it produces large number of disconnected graphs. For selection the conventional crowded binary tournament selection in NSGA-II is used here. As whole subgraph is encoded as a chromosome, so a perturbation in node is performed by means of mutation with a high probability ($p=0.9$).

If a chromosome $n_1,n_2, \ldots , n_9$ is selected to be mutated then the following process is performed:

- Randomly select some of the nodes.
- Insertion and deletion are performed with equal probability:
  - Insertion: Add the nodes which are directly connected with the selected nodes.
  - Deletion: Delete the selected nodes.

**Fig. 1:**
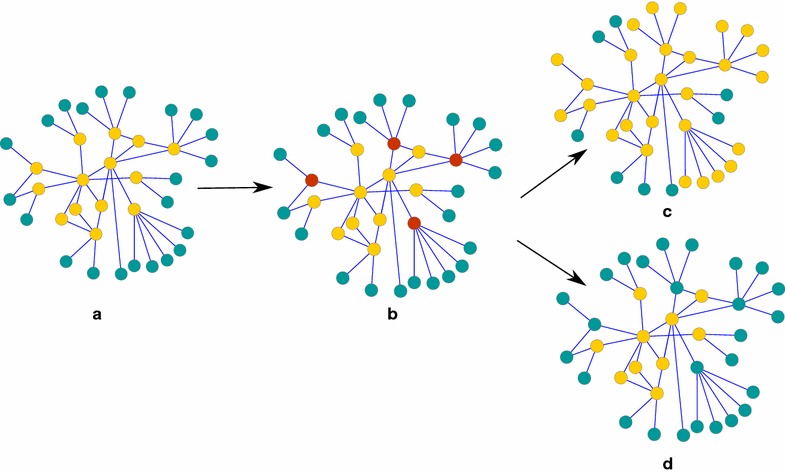
Figure illustrating the mutation procedure. **a** A subgraph in which* yellow nodes* represent chromosome whereas* green nodes* are the first neighbor of these. In **b** the randomly selected* nodes* are colored as *red*. Two process are performed with equal probability: insertion and deletion. **c** and **d** The resulting chromosomes after insertion and deletion operations.

The whole process is shown in Fig. 1.

## Results and discussions

Here we illustrate the performance of our proposed technique and compare this with three well known algorithms MCODE [5], clusterONE [6] and Affinity propagation [7]. We download human PPI dataset from Human Protein Reference Database (HPRD) [17] which contains 39,240 interactions among 9,589 human proteins (The data is given in Additional file 1). Table 1 summarizes the topological properties of the network created from this database. We compare the results with known protein complexes downloaded from a database PCDq [26]. It consists of both predicted and curated human protein complexes and is contains 1,264 complexes with 9,268 proteins and 32,198 PPIs. To investigate the functional enrichment of our predicted complexes we have also performed a Gene Ontology based analysis here. The source code of our proposed method is given in Additional file 2.

**Table 1:** Summary of the human PPI network data sets used here

Data set	#Proteins	#Interactions	Avg. degree	Max. degree	Density	Clustering coefficient	#Connected components	Network diameter	Avg. number of neighbors
HPRD	9,589	39,240	7.924	271	0.001	0.102	262	14	7.703

### Performance comparisons with existing methods

Here, for comparing our results with that of some state-of-the-art algorithms we have utilized some matching statistics like Sensitivity, Positive Predictive Value (PPV), and Accuracy [27].

We have built a Contingency Table (*T*) with *n* rows and *m* columns where rows and columns represent predicted and real protein complexes, respectively. The value of each cell $T_{i,j}$ indicates the number of common proteins between real and predicted complexes.

#### Sensitivity

Sensitivity is defined as the fraction of proteins in real complex *i* found in predicted complex *j*: $Sn_{i,j}=\frac{T_{i,j}}{N_i}$, where $N_i$ is the number of proteins belonging to complex *i*. A complex-wise sensitivity $Sn_{co_i}$ is defined as: $Sn_{co_i}=\max _{j=1}^mSn_{i,j}$. The General Sensitivity ($S_n$) is the weighted average of complex-wise sensitivity $Sn_{co_i}$ over all complexes and is defined as:

4 $$\begin{aligned} S_n=\frac{\sum _{i=1}^nN_iSn_{co_i}}{\sum _{i=1}^nN_i}. \end{aligned}$$

#### Positive predictive value

The positive predictive value is the proportion of proteins in predicted complex *j* which belong to the real complex *i* and is defined as: $PPV_{i,j}=\frac{T_{i,j}}{\sum _{i=1}^nT_{i,j}}=\frac{T_{i,j}}{T_{.j}}$, where $T_{.j}$ is the marginal sum of a column *j*. Complex-wise-wise positive predictive value $PPV_{cl_j}$ represents the maximal fraction of proteins of predicted complex *j* found in some real complex: $PPV_{cl_j}=\max _{i=1}^nPPV_{i,j}$. The General PPV(*PPV*) of a clustering result is the weighted average of complex-wise PPV($PPV_{cl_j}$) over all predicted complexes, and is defined as:

5 $$\begin{aligned} PPV=\frac{\sum _{j=1}^mT_{.j}PPV_{cl_j}}{\sum _{j=1}^mT_{.j}}. \end{aligned}$$

#### Accuracy

The Geometric Accuracy (Acc) represents a tradeoff between sensitivity and the positive predictive value and it is defined as:

6 $$\begin{aligned} Acc=\sqrt{S_n*PPV}. \end{aligned}$$

The advantage of taking the geometric mean is that it yields a low score when either the $S_n$ or the *PPV* metric is low. High accuracy value thus requires a high performance for both the criteria.

In Table 2 we show the comparative performance of different existing algorithms with our proposed method using these three metrics. It may be noted that the proposed method performs comparatively well than the other algorithms with respect to sensitivity, PPV and accuracy.

**Table 2:** Comparison of performance of different algorithms with respect to sensitivity, PPV and accuracy

Method	Sensitivity	PPV	Accuracy
MCODE	0.2134	0.5274	0.3347
ClusterONE	0.1414	0.4562	0.2540
Affinity_propagation	0.1837	0.4443	0.2857
CORE	0.315	0.353	0.333
RNSC	0.379	0.469	0.4216
MCL-Caw	0.331	0.241	0.279
PEWCC	0.435	0.469	0.451
Proposed_Method	0.3061	0.6928	0.4605

We have also performed an analysis to compare the performance of different existing algorithm with the proposed one. Let $B={B_1, B_2, \ldots B_n}$ and $C={C_1, C_2, \ldots C_m}$ be the sets of benchmark and predicted complexes respectively. The Jaccard index *J* represents the overlap between a benchmark complex and predicted complex. It is defined as $J(B_i,C_j)=\frac{|B_i \bigcap C_j|}{|B_i \bigcup C_j|}$. A benchmark complex is said to be covered by a predicted complex if the value of *j* is greater than some threshold value. In this respect Recall (coverage) and precision (sensitivity) can be defined as

7 $$\begin{aligned} Recall=\frac{|B_i|}{|B|}, \end{aligned}$$

where $J(B_i,C_j)>t$, for some $C_j \epsilon C$, and

8 $$\begin{aligned} precision=\frac{|C_j|}{|C|}, \end{aligned}$$

where $J(B_i,C_j)>t$ for some $B_i \epsilon B$

**Fig. 2:**
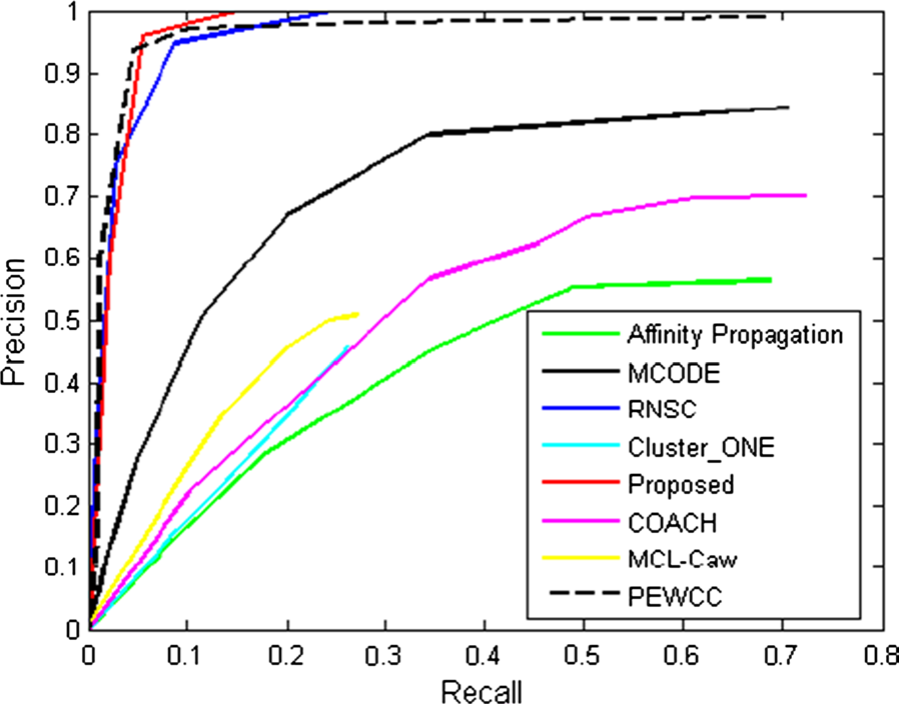
Precision vs recall curves for all methods at different threshold values (t).

We evaluate the performance of the existing methods by plotting the precision versus recall curves for the predicted complexes. These curves are plotted by tuning the threshold value *t* from 0 to 1. This is shown in Fig. 2. From this plot we have also computed the AUC score for each of the methods. The AUC score is shown in Table 3. It is evident from the Fig. 2 and Table 3 that proposed method shows best precision and recall compare to the other state-of-the-art. The processed data for constructing the ROC plot are given in Additional file 3.

**Table 3:** AUC score of different methods

	MCODE	ClusterONE	Affinity_Propagation	COACH	RNSC	MCL-Caw	PEWCC	Proposed method
AUC	0.1096	0.0590	0.2714	0.3572	0.2277	0.0841	0.6595	0.7244

**Table 4:** Predicted protein complexes, their GO-terms, p-values, and KEGG pathways

Sl.No.	Cluster id	Predicted complex (% of proteins covered)	Matched proteins	GO-term (bp)	GO-term (CC)	GO-term (mf)	KEGG pathway
1	5	CTNNB1–DVL1–DVL3–PPM1A complex (80%), HSPB1–PPA1–PPA1–SETDB1–TP53–WIPI1 complex (66.67), EEF1A1–MDH2–WARS complex (66.67), transforming growth factor–SMAD complex (66.67)	DVL2, ‘CTNNB1’ ‘PPM1A’ ; ‘SMAD3’ ‘TP53’ ‘PPA1’ ‘PPA1’; YWHAG’ ‘EEF1A1’; ‘EEF1A1’ ‘SMAD2’	Enzyme linked receptor protein signaling pathway (GO:0007167 ) (5.9E–11)	Cytosol (GO:0005829) (3.4E–11)	Enzyme binding (GO:0019899) (1.5E–12)	Colorectal cancer (2.9E–11), Pathways in cancer(1.2E–10)
2	6	CDH1–CTNNB1–PTPN1 complex(50), transforming growth factor–SMAD complex (66.67), HSPB1–PPA1–PPA1–SETDB1–TP53–WIPI1 complex (66.67)	DVL2 SMAD3 TP53 YWHAG	Response to organic substance (GO:0010033) (4.2E–14)	Cytosol (GO:0005829) (2.5E–25)	Protein kinase activity (GO:0004672) (1.8E–10)	Pathways in cancer (6.1E–19)
3	8	MARCKS–NMT1–TP53 complex (66.67), transforming growth factor–SMAD complex (75), CTNNB1–DVL1–DVL3–PPM1A complex (50)	CTNNB1 SMAD3 TP53 YWHAG TP53 SMAD2	Protein amino acid phosphorylation (GO:0006468) (4.1E–14)	Cytosol (GO:0005829) (3.8E–23)	Protein serine/threonine kinase activity (GO:0004674) (1.7E–11)	Chronic myeloid leukemia (5.7E–16)
4	11	p300–MDM2–p53 protein complex (63.64), CDH1–CTNNB1–PTPN1 complex (60)	HDAC1 TP53 ESR1 TP53 CTNNB1	Positive regulation of nitrogen compound metabolic process (GO:0051173) (4.1E–20)	Nucleoplasm (GO:0005654) (2.6E–18)	Transcription factor binding (GO:0008134) (1.5E–15)	Prostate cancer (2.0E–12)
5	15	p300–MDM2–p53 protein complex (72.73), CASP8–MAPK1–MAPK3–PEA15–RPS6KA3 complex (60),CDH1–CTNNB1–PTPN1 complex (75), EP300–HOXB6–HOXB7 complex (66.67)	EP300 HDAC1 TP53 ESR1 LCK HSP90AA1 MAPK1 MAPK3 EP300 TP53 EP300 TP53 FOXO1 SMAD4 EP300 SMAD2 CTNNB1 PTPN1 HGS EP300 EEF1A1 SMAD2	Positive regulation of macromolecule metabolic process (GO:0010604) (4.5E–34)	Nucleoplasm (GO:0005654) (3.1E–34)	Transcription regulator activity (GO:0030528) (7.6E–26)	Chronic myeloid leukemia (9.1E–26)
6	20	Smad protein complex (53.33), FOXO–SMAD complex (60), transforming growth factor–SMAD complex (75), CDH1–CTNNB1–PTPN1 complex (75)	SMAD3 SMAD2 LCK HSP90AA1 TP53 YWHAG CTNNB1 SMAD2	Positive regulation of cellular biosynthetic process (GO:0031328) (6.8E–23)	Cytosol (GO:0005829) (3.9E–15)	Enzyme binding (GO:0019899) (1.5E–20)	Adherens junction (1.2E–16)
7	25	EEF1A1–MDH2–WARS complex (66.67), CTNNB1–DVL1–DVL3–PPM1A complex (66.67)	DVL2 CTNNB1 PPM1A SMAD3 TP53 PPA1 PPA1 YWHAG EEF1A1 EEF1A1 SMAD2	Enzyme linked receptor protein signaling pathway (GO: 0007167) (2.5E–15)	Cytosol (GO:0005829) (6.1E–20)	SMAD binding (2.2E–13) (GO:0046332)	Pathways in cancer (3.5E–21)
8	26	CDK5R2–CHN1–ERBB2 complex (66.67), EEF1A1–MDH2–WARS complex(66.67)	SMAD3 TP53 YWHAG SMAD2	Response to organic substance (GO:0010033) (4.9E–12)	Cytosol (GO:0005829) (3.9E–20)	Enzyme binding (GO:0019899) (8.0E–16)	Colorectal cancer(6.4E–14)
9	36	SMAD1–SMAD4–ECSIT2 containing complex (80), CDH1–CTNNB1–PTPN1 complex (75), p300–MDM2–p53 protein complex (81.82)	‘BRCA1’ ‘CTNNB1’ ‘ECSIT’ ‘EP300’ ‘ESR1’ ‘HDAC1’ ‘MDM2’ ‘PTPN1’ ‘RB1’ ‘SMAD1’ ‘SMAD4’ ‘SP1’ ‘TP53’ ‘UBE2Z’	Positive regulation of cellular biosynthetic process (GO:0031328) (7.8E–37)	Nuclear lumen (GO:0031981) (3.0E–27)	Transcription regulator activity (1.7E–30) (GO:0030528)	Pathways in cancer(2.6E–30)
10	39	FOXO–SMAD complex (60), EEF1A1–MDH2–WARS complex (66.67), CDH1–CTNNB1–PTPN1 complex (75)	‘CDH2’ ‘CTNNB1’ ‘EEF1A1’ ‘FOXO1’ ‘FOXO3’ ‘SMAD1’ ‘SMAD4’ ‘YWHAG’	Cell cycle (GO:0007049) (1.2E–30)	Nucleoplasm (GO:0005654) (8.8E–59)	Transcription factor binding (GO:0008134) (1.5E–25)	Cell cycle (1.6E–18)
11	40	ERBB2IP–ZFYVE9 containing complex, p300–MDM2–p53 protein complex (72.73), CDK7–cyclin H complex (60)	‘AR’ ‘BRCA1’ ‘CDK2’ ‘EP300’ ‘ERBB2IP’ ‘ESR1’ ‘HDAC1’ ‘MDM2’ ‘MNAT1’ ‘SP1’ ‘TBP’ ‘TP53’ ‘UBE2I’	Positive regulation of macromolecule metabolic process (GO:0010604) (1.6E–29)	Nuclear lumen(GO:0031981) (1.3E–19)	Transcription activator activity (GO:0016563) (3.2E–14)	Pathways in cancer(3.3E–16)
12	10	CDK7–cyclin H complex (80), CASP8–MAPK1–MAPK3–PEA15–RPS6KA3 complex (80), ITPR1–STARD13–TXNDC4 complex (66.67), PIN1–TP53 complex(66.67)	‘AR’ ‘CASP8’ ‘CDK2’ ‘CDK7’ ‘HMGB1’ ‘ITPR1’ MAPK1’ ‘MAPK3’ ‘MDM2’ ‘MNAT1’ ‘PEA15’ ‘PIN1’ ‘TP53’ ‘TP73’	Positive regulation of gene expression (GO:0010628) (1.0E–23)	Transcription factor complex (GO:0005667) (9.8E–13)	Enzyme binding (GO:0019899) (9.7E–21)	Pathways in cancer (7.0E–26)
13	51	HSPA1A–TP53 complex (100), ATXN1–C1orf94–DAZAP2–RBPMS–UBQLN4 containing complex (66.67), p300–MDM2–p53 protein complex (80)	‘ATXN1’ ‘BAT2’ ‘BRCA1’ ‘DAZAP2’ ‘EP300’ ‘ERBB2IP’ ‘ESR1’ ‘HDAC1’ ‘HSPA1A’ ‘MDM2’ ‘SP1’ ‘TBP’ ‘TP53’ ‘UBE2I’ ‘UBQLN4’	Positive regulation of cellular biosynthetic process (GO:0031328) (4.7E–32)	Nucleoplasm (GO:0005654) (1.7E–28)	Transcription regulator activity (GO:0030528) (3.2E–20)	Pathways in cancer (4.2 E–24)
14	3	CDH1–CTNNB1–PTPN1 complex (75), EEF1A1–MDH2–WARS complex(66.67)	‘CDH2’ ‘CTNNB1’ ‘EEF1A1’ ‘PTPN1’ ‘YWHAG’	Positive regulation of macromolecule metabolic process (GO:0010604) (8.7E–28)	Nucleoplasm (GO:0005654) (2.0E–18)	Transcription factor binding (GO:0008134) (1.3E–18)	Neurotrophin signaling pathway (2.3E–12)
15	38	COP9 signalosome (CSN) (70), mutant p53/NF-Y protein (mutp53/NF-Y) complex (66.67), TP53–TP73 complex (66.67), HSPB1–PPA1–PPA1–SETDB1–TP53–WIPI1 complex (66.67)	‘COPS3’ ‘COPS4’ ‘COPS5’ ‘COPS6’ ‘COPS7A’ ‘COPS8’ ‘CREBBP’ ‘EP300’ ‘GPS1’ ‘NFYA’ ‘PPA1’ ‘SMAD3’ ‘SP1’ ‘TP53’ ‘TP73’ ‘WT1’	Positive regulation of nitrogen compound metabolic process (GO:0051173) (6.9E–22)	Cytosol (GO:0005829) (1.4E–10)	Enzyme binding (GO:0019899) (2.8E–18)	Pathways in cancer (7.0E–20)
16	21	MDM2–PML–PML–SUMO1–SUZ12 complex (80), SUMO1 activation complex (66.67), p300–MDM2–p53 protein complex (80)	‘AR’ ‘BRCA1’ ‘DAXX’ ‘EP300’ ‘ESR1’ ‘HDAC1’ ‘MDM2’ ‘PIAS1’ ‘PML’ ‘RB1’ ‘SP1’ ‘SUMO1’ ‘TP53’ ‘UBE2I’	Positive regulation of transcription (5.7E–34)	Organelle lumen (GO:0043233) (8.3E–34)	Transcription regulator activity (GO:0030528) (4.9E–39)	Pathways in cancer (3.9E–21)
17	30	CDH1–CTNNB1–PTPN1 complex (75), PKD1–PKD2 complex (66.67), EEF1A1–MDH2–WARS complex (66.67)	‘CDH2’ ‘CTNNB1’ ‘EEF1A1’ ‘PKD1’ ‘PTPN1’ ‘YWHAG’	Positive regulation of nitrogen compound metabolic process (GO:0051173) (1.0E–17)	Cell projection (GO:0042995) (9.0E–8)	Enzyme binding (GO:0019899) (2.1E–13)	Adherens junction (1.5E–18)
18	13	TP53–TP73 complex (66.67), p300–MDM2–p-53 protein complex (72.73)	‘BRCA1’ ‘CREBBP’ ‘EP300’ ‘ESR1’ ‘HDAC1’ ‘MDM2’ ‘SP1’ ‘TBP’ ‘TP53’ ‘TP73’ ‘WT1’	Positive regulation of cellular biosynthetic process (GO:0031328) (3.1E–25)	Nuclear lumen (7.3E–24)	Transcription regulator activity (GO:0030528) (2.2E–24)	Prostate cancer (8.5E–9)

**Fig. 3:**
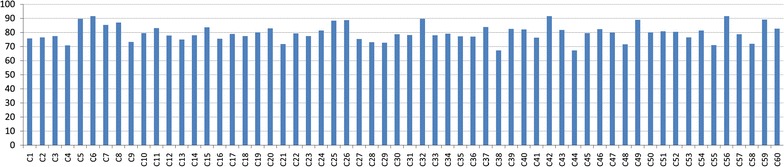
Bar diagram showing the proportion of proteins in predicted clusters that are involved in some real protein complexes.

### Analysis of predicted complexes

In Table 4 we have shown the resulting protein complexes and compared them against the real one. Most of them show a good overlap with real complexes. We plot a bar diagram to show this overlap. In Fig. 3 Y-axis represents proportion of proteins in predicted complexes involved in some real one. From this figure it is noticeable that most of the protein complexes share good proportion of proteins with some real complexes with an average proportion of 79.68%. The smallest complex consists of 11 proteins in which 8 proteins are involved in some real complexes whereas the largest one comprises of 272 proteins out of which 183 proteins are involved in some real complexes. We have also preformed a GO and pathway based study to biologically validate the predicted complexes.

**Table 5:** Some useful metrics of disorder associated genes

Disease category	No. of associated genes	No. of interaction in human PPI	Density	Semantic similarity
Bone	180	94	0.0163	0.5676
Cancer	869	725	0.0019	0.8931
Cardiovascular	332	457	0.0083	0.7374
Connective_tissue	149	75	0.0068	0.7146
Dermatological	270	154	0.0042	0.6381
Developmental	143	219	0.0216	0.7589
Ear, nose, throat	177	183	0.0117	0.8277
Endocrine	305	572	0.0123	0.8432
Gastrointestinal	110	52	0.0087	0.5819
Hematological	389	755	0.0100	0.6449
Immunological	290	658	0.0157	0.8159
Metabolic	638	2,088	0.0103	0.7458
Muscular	294	1,183	0.0275	0.6421
Neurological	917	898	0.0021	0.7811
Nutritional	51	82	0.0643	0.6536
Ophthalmological	563	555	0.0037	0.7035
Psychiatric	75	49	0.0177	0.7032
Renal	170	131	0.0091	0.6545
Respiratory	79	1	0.00032	0.7023
Skeletal	278	186	0.0048	0.6679
Unclassified	64	6	0.0030	0.5080
Multiple	721	427	0.0016	0.8019

**Fig. 4:**
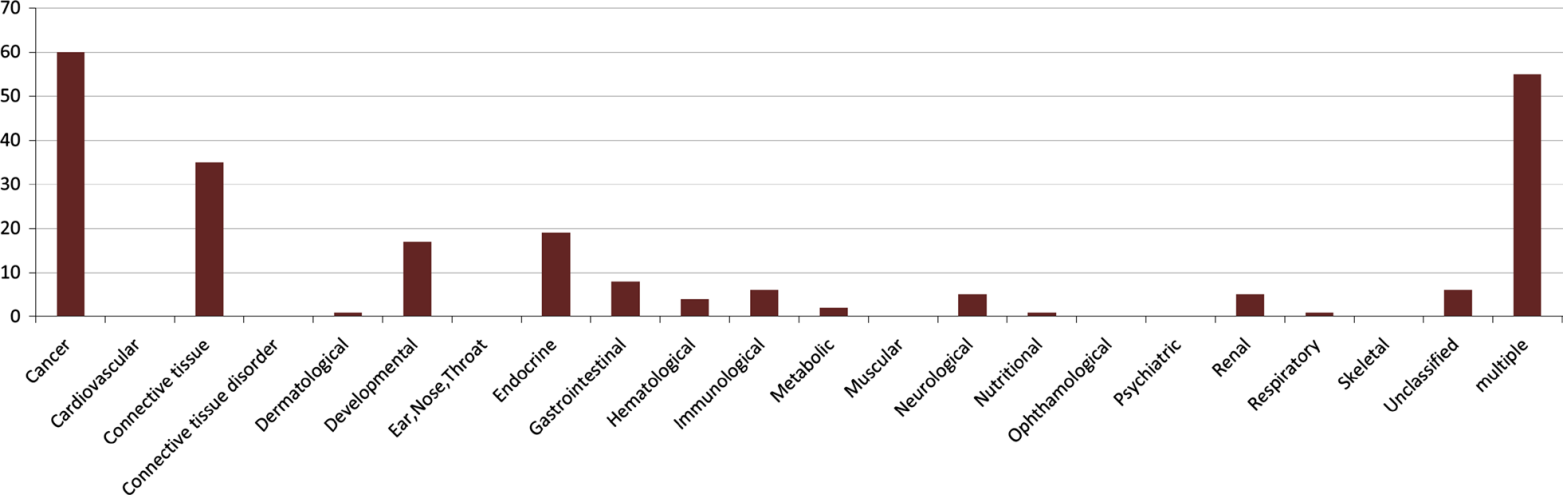
Bar diagram showing the involvement of predicted complexes in 22 primary disease classes.

**Fig. 5:**
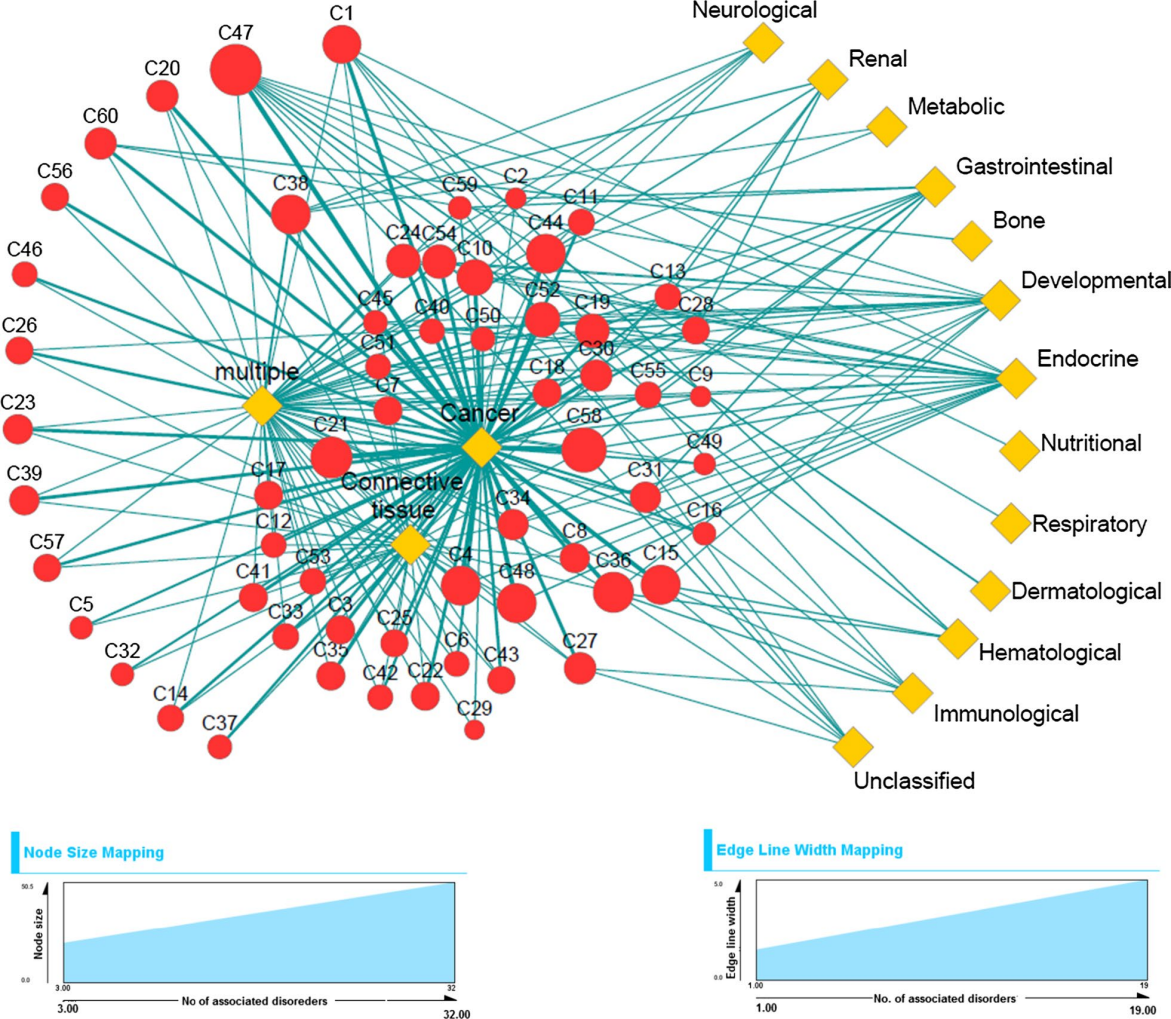
Bipartite network showing the association of predicted complexes and 16 disease classes. The* red nodes* represent predicted complexes and* yellow diamond shaped nodes* denote disease classes. Size of complex nodes are varied according to the number of associated disorders involved with that complex. *Edge width* indicates the number of associated disorders between complex and disease node linked by that edge.

Columns 3 and 4 of Table 4 represent predicted complexes and the list of proteins that are matched with some real one, respectively. Columns 5, 6, 7 and 8 of this table represent most significant GO-terms with three annotations [viz., biological process (BP), cellular component (CC), molecular function (MF)] and KEGG pathways that are associated with the predicted complexes. Here, we notice that more than one complex are grouped in one predicted cluster. For example in row 4 of Table 4 the predicted cluster captured two complexes: p300–MDM2–p53 protein complex and CDH1–CTNNB1–PTPN1 complex. It is not quite unexpected because the real complexes that have some common functionality or have some common signaling pathway tend to group in one cluster. In row 1, predicted cluster 5 captures good proportion proteins of four real complexes viz., CTNNB1–DVL1–DVL3–PPM1A complex (80%), HSPB1–PPA1–PPA1–SETDB1–TP53–WIPI1 complex (66.67%), EEF1A1–MDH2–WARS complex (66.67%) and transforming growth factor–SMAD complex (66.67%). The complex CTNNB1–DVL1–DVL3–PPM1A is composed of genes DVL1, DVL2, CTNNB1, PPM1A and DVL3. Protein phosphatase 1A (PPM1A) is an enzyme which is encoded by the PPM1A gene. The proteins encoded by this gene is a member of PP2C family of Ser/Thr protein phosphatases and are generally known to be a negative regulator of cell stress response pathways. Catenin (cadherin-associated protein), beta 1 (CTNNB1) is an integral part of the canonical Wnt signaling pathway which is a network of proteins that passes signals from receptor of the surface of cell to the nucleus that leads to the expression of target genes. Signaling via Wnt signaling pathway also causes activation of histone methyltransferase (SETDB1) and subsequently represses PPARgamma transactivation [28]. Moreover SMAD1 indirectly enhances Wnt signaling by suppressing the expression of Wnt signaling inhibitors [Dickkopf 1 (Dkk1) and 2] with interleukin (IL)-11. We can notice from Table 4, row-1 that predicted cluster 5 is associated with Colorectal cancer pathway (p-value: 2.9E$-$11). In different literature [29–31] it is established that activation of the Wnt signaling pathway via mutation of the adenomatous polyposis coli gene (APC) is the critical reason for colon carcinogenesis.

Most of our predicted protein complexes are associated with SMAD complexes like transforming growth factor–SMAD complex, FOXO–SMAD complex, SMAD1–SMAD4–ECSIT2 containing complex etc. SMADs are intracellular proteins that transduce extracellular signals from transforming growth factor beta (TGF-$\beta$) ligands to the nucleus. In the nucleus, SMAD complexes attach in some specific areas of DNA and control the activity of particular genes and regulate cell proliferation [32].

From Table 4 it is worth-mentioning that most of the predicted clusters are enriched with several cancer related pathways viz., colorectal cancer, chronic myeloid leukemia, prostate cancer etc. This suggests that the predicted clusters are biologically meaningful and important for uncovering different cancer associated modules.

### Association of predicted complexes in disorders/diseases

Here, we discuss the involvement of predicted protein complexes in different disease/disorder classes. The list of disorders/diseases, disorder associated genes and association between genes and disorder/disease classes are obtained from Goh et al. [23]. In Goh et al., a classification of disorders can be found depending on the physiological system affected by the disorder. They have classified all genetic disorders in 22 primary classes and associated all the genes corresponding to all the disorders. In each class there is a list of disorders/diseases that exhibit similar type of clinical features affected by these disorders. Here, we find an association of our predicted complexes with these 22 disorder/disease classes. To test the biological plausibility of the identified complexes we draw a bipartite network between protein complexes and 22 disorders/diseases to find disease associated complexes. We have also tested the involvement of proteins belonging to our predicted complexes in those disorders.

#### Involvement of identified complexes in 22 primary disorders/disease classes

To show to what extent the proteins of our predicted complexes are involved in specific disorder/disease classes we plot a bar diagram showing the proportion of protein complexes involved in each of the disorder classes. We assume that a protein complex is associated with specific type of disorder if all the proteins associated with this disorder are belonging to that protein complex. The bar diagram is shown in Fig. 4. From this figure we can notice that subsequent number of protein complexes are associated with ‘cancer’ and ‘multiple’ disease classes. ‘Cancer’ class consists of 113 disorders whereas ‘multiple’ class contains 155 disorders. The disorders are assigned in each class based on the similarity of clinical properties of these disorders and the observation of physiological system mostly affected by those disorders. Disorders having multiple clinical features are placed in the ‘multiple’ class.

**Fig. 6:**
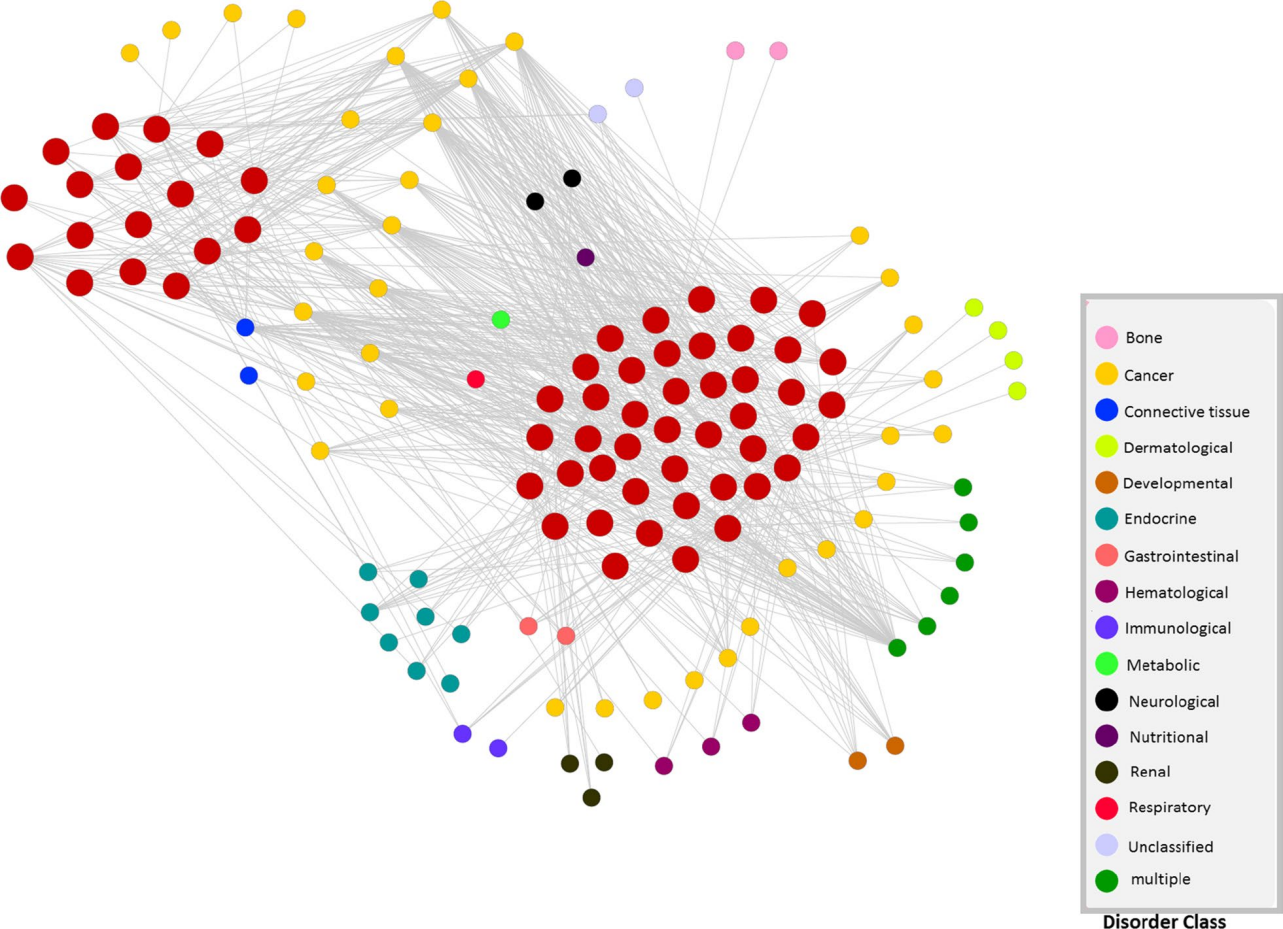
Bipartite network showing direct association between predicted complexes and disorders. The* big red node* represent predicted complexes whereas* small nodes* denote different disorders. Disorder* nodes* are *colored* according to the involvement of associated disease classes.

We observe from Fig. 4 that most of the predicted complexes are enriched with proteins that are implicated by different disorders involved with ‘cancer’ and ‘multiple’ disorder classes. Some proteins of our predicted complexes are also involved in the disorders associated with ‘connective tissue’, ‘Developmental’ and ‘Endocrine’ classes. We have been able to associate the predicted complexes with 15 classes of disorders amongst 22 disorder classes.

#### Complex–disease bipartite network

To identify the overall association between predicted complexes and disease classes we have created a bipartite network between predicted protein complexes and the associated disease classes. One partite set constitutes the complexes, whereas other partite set represents the associated disease classes. Each partite set is connected with other by edges depending on the association between predicted complexes and the disorders involved in disease classes. The network is shown in Fig. 5. The predicted complexes are represented by red nodes whereas the disease classes are denoted by yellow diamond shaped nodes. It is possible that several proteins in one complex are involved in several disorders belonging to different disease classes. So, we have calculated the number of disorders associated with each predicted complex. We say a disorder is associated with a predicted complex if all the proteins associated with that disorder is involved in that complex. To show to what extent the protein complexes are associated with disorders we vary the size of red nodes based on the total number of disorders associated with those complexes. An edge between a complex and a disease class indicates the association of disorders belonging to the disease class with that complex. Edge width indicates the number of associated disorders with the corresponding complex and disease class linked by that edge. It appears from Fig. 5 that most of the complexes are associated with significant number of disorders belonging to different disease classes. Interestingly it is found that all the complexes are more or less associated with ‘cancer’ related disorders. It suggests that the predicted complexes are enriched with proteins that are involved in different cancer related disorders and may be considered as important candidates to uncover different associations for understanding disease mechanisms, diagnosis and therapy. Other disorders like ‘Connective tissue’, ‘multiple’ and ‘Endocrine’ also show reasonable amount of association with different complexes. In Additional file 3 the processed data for constructing the complex–disease bipartite network are given.

We have also performed an analysis to describe the association among protein complexes and disease classes. For this purpose, we have collected PPI information and GO based semantic similarity information of all the genes associated with 22 disease classes. The PPI and GO semantic similarity informations are subsequently converted into 22 PPI and GO semantic similarity networks.

We have computed density and average semantic similarity scores from these networks and showed in Table 5. First and second column of the table represent disease category and number of associated genes in it, respectively. The third column shows number interactions among the genes, in each category. The last two columns represent density and average semantic similarity score of interaction network. It is noticeable that among all disease classes cancer associated proteins have better score than others. The predicted complexes are functionally similar, and it may be a possible reason for the over-representation of cancer associated genes in predicted complexes. For the similar reason, other disorders like ‘Connective tissue’, ‘multiple’ and ‘Endocrine’ have better amount of association with different complexes.

To identify the association among disorders and protein complexes, we have also created another bi-partite network. As depicted in Fig. 6, the network is composed of two types of nodes. The big red nodes represent predicted complexes and small nodes stand for different disorders. Out of 22 disease classes we have found 16 classes have different disorders associated with the predicted complexes. In Additional file 4: Table S1 we have listed all the disorder names associated with specific complexes. 1st column of the Table S1 (Additional file 4) represents predicted protein complex, whereas the second and third columns represent the associated disorders and the corresponding disease class. From this table we can notice that the proteins in complex 1 is associated with 11 ‘cancer’ associated disorders, 3 hematological disorders, 1 ‘Endocrine’, ‘Connective tissue’, and ‘Immunology’ related disorders. All the proteins implicated by those disorders are clustered in complex 1. This suggests that these associated disorders of different disease classes are loosely related with each other. For example ‘Leukemia, acute promyelocytic disorder’ may be developed by a long course of ‘Polycythemia vera’ which is associated with ‘Hematological’ disorders [33, 34]. From the Table S1 (Additional file 4) we can notice that the disorder ‘Pilomatricoma’ which belongs to the ‘Cancer’ disease class and the disorder ‘Rubinstein–Taybi syndrome’ related to the ‘multiple’ disease class are involved in complex 1. In [35] an abnormal association between multiple perforating and non-perforating pilomatricomas with Rubinstein–Taybi syndrome are reported. From Table S1 (Additional file 4) it can also be observed that the disorder ‘Thrombocythemia, essential’ involved in ‘Hematology’ disease class is grouped with disorder ‘Renal cell carcinoma’ belonging to ‘Cancer’ disease class in complex 1. This suggests that these two disorders are somehow related based on topological and ontological properties of the proteins that are directly associated with those disorders. It is because all the proteins implicated by these two disorders are grouped in the same complex. Interestingly in [36] a statistical correlation is also observed between essential Thrombocythemia and the survival of surgically treated renal cell carcinoma patients.

From Table S1 (Additional file 4) we can see that complex 4 is associated with 12 cancer related disorders, 3 endocrine disorders and 1 multiple disorder. The predicted complex C4 contains all the proteins which are related with ‘Hyperparathyroidism’ disorder and ‘Colorectal Cancer’ disorder. ‘Hyperparathyroidism’ is a disorder in which parathyroid (PTH) glands are overactivated and produces excess PTH hormone in our body. It is reported that primary hyperparathyroidism (PHP) is associated with malignancy and decreased intracolonic calcium (Ca) that plays a role in colorectal carcinogenesis [37]. We can notice that disorder ‘Parathyroid adenoma’ is also associated with complex C4. A ‘parathyroid adenoma’ is a noncancerous (benign) tumor of the parathyroid glands but in many cases it is reported that it causes ‘Hyperparathyroidism’ [38, 39]. ‘Rabson–Mendenhall syndrome’ in ‘multiple’ disease class is a rare genetic disorder mainly caused by mutation of insulin receptor gene ‘INSR’. It appears from the Table S1 (Additional file 4) that this disorder is associated with about eight predicted complexes. Most of these associated complexes also contain other disorders which are belonging to different disease classes and are linked with extreme insulin resistance due to mutations in the insulin receptor gene ‘INSR’. For example complex C7 is associated with disorder ‘Leprechaunism’ [commonly known as Donohue syndrome (OMIM 246200)] which is a latent inherited disorder and is caused by defect of insulin receptor genes. This suggests that although the disorders ‘Rabson–Mendenhall syndrome’ and ‘Leprechaunism’ are belonging to different disease classes but the genes responsible for these disorders exhibit substantial amount of similar functional information. The possible reason behind this is that these two disorders show strong inclination in being grouped in most of the complexes.

In most of the cases we observe that similar type of disorders have a tendency to get involve in same protein complexes. So we can conclude that protein complexes not only provide a better understanding in molecular evolution but it can also unveil several information of human disorders and uncover new strategies for therapeutic intervention. This may lead to development of new potential strategies to deal with key diseases by giving more importance in protein complex formation information rather than targeting individual proteins.

## Conclusions

This study introduces a multiobjective approach for detection of protein complexes in human PPI network. Integrating topological features along with GO features, we are able to group functionally similar proteins in same clusters which serve as protein complexes. The algorithm progresses with two primary classes of objectives. Graph based objectives preserve the topological properties of complexes whereas GO based semantic similarity between protein pairs control the accumulation of functionally similar proteins in the same cluster. Moreover the predicted complexes show consistently better result in context of some performance metrics.

We have also built an association between predicted protein complexes and 22 primary key disease classes to study the relationship between complexes and disorders associated with these classes. For finding the association of predicted complexes in different disease classes, we have extensively searched the involvement of proteins implicated by different disorders in the predicted complexes and built two bipartite networks between complexes and disorders. Interestingly, we have found most of the predicted complexes are associated with disorders belonging to the ‘Cancer’ disease class. Additionally, the disorders belonging to ‘Endocrine’ and ‘multiple’ disease classes have also shared a significant proportion of proteins involved in multiple predicted complexes.

Protein complexes are now considered as potential targets for intervention of new therapeutics to treat against new diseases. It is possible to integrate the drug–disease association information along with the complex–disease association. The new paradigm in drug discovery analysis is now given emphasis on the polypharmacological properties of drugs. Polypharmacological drugs are generally targets multiple cellular function for the treatment of complex diseases. By incorporating protein complexes with drug–disease association, it may be possible to uncover some relationship between protein complexes and targeted drugs.

Moreover we can potentially merge the time series gene expression profiles affected by a specific disease with our complex–disease bipartite network structure. This can offer a new way to exploit new topological features and complex modular structure in protein complex–disease and protein complex–disease–drug network. We are now working in this direction.

## Additional files

Additional file 1. The human protein–protein interaction data. Additional file 2. The source code of the proposed methodology. Additional file 3. The processed data for constructing complex-disease bipartite network and ROC plot. Additional file 4: Table S1. The predicted protein complexes and their association with multiple disorders corresponding to the specific disease classes.
