# Supplementary material for: A multiobjective approach for identifying protein complexes and studying their association in multiple disorders
Source: Algorithms Mol Biol. 2015 Aug 9;10:24. doi: 10.1186/s13015-015-0056-2 (PMC4529733; doi:10.1186/s13015-015-0056-2)
Supplement: Additional file 2. — The source code of the proposed methodology. [file 13015_2015_56_MOESM2_ESM.zip › code/readme_updated.docx]

One sample run of main function and its outputs.

[front,Result_Pop] = MOEA_PPIN_Biological (50,5,adj,shortest_path_matrix,Similarity_Matrix)

Similarity Matrix

Population Size

Shortest path distance Matrix

Adjacency Matrix

No. of generation

Resulting Population

OUTPUT

******************************************************************************************************

Result_Pop {i} i=1,2,………..50, represent i^th^ cluster that contains indexes corresponding to the names of proteins.

******************************************************************************************************

Result_Pop = Columns 1 through 7

[1x21 double] [1x89 double] [1x20 double] [1x25 double] [1x144 double] [1x50 double] [1x144 double]

Columns 8 through 14

[1x27 double] [1x26 double] [1x213 double] [1x62 double] [1x48 double] [1x27 double] [1x65 double]

Columns 15 through 20

[1x136 double] [1x42 double] [1x147 double] [1x127 double] [1x32 double] [1x49 double]

Columns 21 through 26

[1x140 double] [1x295 double] [1x103 double] [1x105 double] [1x33 double] [1x136 double]

Columns 27 through 32

[1x116 double] [1x116 double] [1x23 double] [1x127 double] [1x325 double] [1x41 double]

Columns 33 through 39

[1x95 double] [1x19 double] [1x89 double] [1x23 double] [1x143 double] [1x77 double] [1x55 double]

Columns 40 through 45

[1x63 double] [1x149 double] [1x67 double] [1x21 double] [1x146 double] [1x43 double]

Columns 46 through 50

[1x131 double] [1x70 double] [1x55 double] [1x123 double] [1x152 double]
