# Supplementary material for: A multiobjective approach for identifying protein complexes and studying their association in multiple disorders
Source: Algorithms Mol Biol. 2015 Aug 9;10:24. doi: 10.1186/s13015-015-0056-2 (PMC4529733; doi:10.1186/s13015-015-0056-2)
Supplement: Additional file 4: Table S1. — The predicted protein complexes and their association with multiple disorders corresponding to the specific disease classes. [file 13015_2015_56_MOESM4_ESM.pdf]

Supplementary table: Predicted protein complexes and their association with multiple disorders corresponding to specific disease classes

Protein Complexes	Associated Disorders	Disease Class
C1	Adenocarcinoma of lung, response to tyrosine kinase inhibitor in, ;Colorectal cancer ;Hepatoblastoma ;Hepatocellular carcinoma, ;Hepatocellular carcinoma, childhood type, ;Leukemia, acute promyeloyctic, STATB/RARA type ;Nonsmall cell lung cancer, response to tyrosine kinase inhibitor in, ;Ovarian carcinoma, endometrioid type ;Pilomatricoma, ;Renal cell carcinoma, papillary, familial and sporadic, ;Squamous cell carcinoma, head and neck, ;	Cancer
	Loeys-Dietz syndrome, ;	Connective tissue
	Growth hormone insensitivity with immunodeficiency, ;	Endocrine
	Myelofibrosis, idiopathic, ;Polycythemia vera, ;Thrombocythemia, essential, ;	Hematological
	Mycobacterial infection, atypical, familial disseminated, ;	Immunological
	STAT deficiency, complete ;	Unclassified
	Rubinstein-Taybi syndrome, ;	multiple
C2	Colorectal cancer, ;	Cancer
	Cirrhosis, cryptogenic ;Cirrhosis, noncryptogenic, susceptibility to, ;	Gastrointestinal
	Rubinstein-Taybi syndrome, ;	multiple
C3	Adenocarcinoma of lung, response to tyrosine kinase inhibitor in, ; Colorectal cancer ;Hepatoblastoma ;Hepatocellular carcinoma, ;Nonsmall cell lung cancer, response to tyrosine kinase inhibitor in, ;Ovarian carcinoma, endometrioid type ;Pilomatricoma, ;Squamous cell carcinoma, head and neck, ;	Cancer
	Loeys-Dietz syndrome, ;	Connective tissue
	Rubinstein-Taybi syndrome, ;	multiple
C4	Accelerated tumor formation, susceptibility to ;Adrenal adenoma, sporadic ;Breast cancer ;Burkitt lymphoma, ;Carcinoid tumor of lung ;Colorectal cancer, ;Hepatoblastoma ;Hepatocellular carcinoma, ;Leukemia, Philadelphia chromosome-positive, resistant to imatinib ;Lipoma, sporadic ;Multiple endocrine neoplasia I ;Ovarian carcinoma, endometrioid type ;Parathyroid adenoma, sporadic ;Pilomatricoma, ;Squamous cell carcinoma, head and neck, ;	Cancer
	Loeys-Dietz syndrome, ;	Connective tissue
	Angiofibroma, sporadic ;Hyperparathyroidism, AD, ;Prolactinoma, hyperparathyroidism, carcinoid syndrome ;	Endocrine
	Rubinstein-Taybi syndrome, ;	multiple
C5	Colorectal cancer ;Hepatoblastoma ;Hepatocellular carcinoma, ;Ovarian carcinoma, endometrioid type ;Pilomatricoma, ;	Cancer
	Loeys-Dietz syndrome, ;	Connective tissue

C6	Colorectal cancer, ; Hepatoblastoma ;Hepatocellular carcinoma, ;Ovarian carcinoma, endometrioid type ;Pilomatricoma, ;	Cancer
	Loeys-Dietz syndrome, ;	Connective tissue
	Rubinstein-Taybi syndrome, ;	multiple
C7	Colorectal cancer ;Hepatoblastoma ;Hepatocellular carcinoma, ;Ovarian carcinoma, endometrioid type ;Pilomatricoma, ;	Cancer
	Loeys-Dietz syndrome, ;	Connective tissue
	Leprechaunism, ;	Developmental
	Diabetes mellitus, insulin-resistant, with acanthosis nigricans ;	Endocrine
	Rabson-Mendenhall syndrome, ;Rubinstein-Taybi syndrome, ;	multiple
C8	Adenocarcinoma of lung, somatic, ;Colorectal cancer ;Colorectal cancer, somatic ;Hepatoblastoma ;Hepatocellular carcinoma, ;Leukemia, Philadelphia chromosome-positive, resistant to imatinib ;Melanoma, melignant, somatic ;Nonsmall cell lung cancer, somatic ;Ovarian carcinoma, endometrioid type ;Pilomatricoma, ;	Cancer
	Loeys-Dietz syndrome, ;	Connective tissue
	Diabetes mellitus, noninsulin-dependent ;	Endocrine
C9	Colorectal cancer, ;Leukemia, Philadelphia chromosome-positive, resistant to imatinib ;Squamous cell carcinoma, head and neck, ;	Cancer
	Rubinstein-Taybi syndrome, ;	multiple
C10	Accelerated tumor formation, susceptibility to ;Adenocarcinoma of lung, response to tyrosine kinase inhibitor in, ;Bladder cancer, ;Burkitt lymphoma, ;Colorectal cancer ;Hepatoblastoma ;Hepatocellular carcinoma, ;Leukemia/lymphoma, B-cell, ;Nonsmall cell lung cancer, response to tyrosine kinase inhibitor in, ;Ovarian carcinoma, endometrioid type ;Pilomatricoma, ;Retinoblastoma ;	Cancer
	Leprechaunism, ;	Developmental
	Diabetes mellitus, insulin-resistant, with acanthosis nigricans ;	Endocrine
	Amyloidosis, Finnish type, ;	Neurological
	Rabson-Mendenhall syndrome, ;Rubinstein-Taybi syndrome, ;	multiple
C11	Accelerated tumor formation, susceptibility to ;Colorectal cancer ;Hepatoblastoma ;Hepatocellular carcinoma, ;Leukemia, acute myeloblastic ;Ovarian carcinoma, endometrioid type ;Pilomatricoma, ;	Cancer
	Rubinstein-Taybi syndrome, ;	multiple
C12	Colorectal cancer ;Hepatoblastoma ;Hepatocellular carcinoma, ;Ovarian carcinoma, endometrioid type ;Pilomatricoma, ;	Cancer
	Loeys-Dietz syndrome, ;	Connective tissue
	Rubinstein-Taybi syndrome, ;	multiple

C13	Accelerated tumor formation, susceptibility to ;Colorectal cancer, ;Squamous cell carcinoma, head and neck, ;Wilms tumor, type , ;	Cancer
	Denys-Drash syndrome, ;Mesangial sclerosis, isolated diffuse, ;	Renal
	Frasier syndrome, ;Rubinstein-Taybi syndrome, ;WAGR syndrome, ;	multiple
C14	Colorectal cancer ;Hepatoblastoma ;Hepatocellular carcinoma, ;Ovarian carcinoma, endometrioid type ;Pilomatricoma, ;Squamous cell carcinoma, head and neck, ;	Cancer
	Loeys-Dietz syndrome, ;	Connective tissue
	Rubinstein-Taybi syndrome, ;	multiple
C15	Accelerated tumor formation, susceptibility to ;Adenocarcinoma of lung, response to tyrosine kinase inhibitor in, ;Bladder cancer, ;Burkitt lymphoma, ;Colorectal cancer ;Hemangioma, capillary infantile, somatic, ;Hepatoblastoma ;Hepatocellular carcinoma, ;Leukemia, acute myeloblastic ;Nonsmall cell lung cancer, response to tyrosine kinase inhibitor in, ;Ovarian carcinoma, endometrioid type ;Pilomatricoma, ;Retinoblastoma ;	Cancer
	Intrauterine and postnatal growth retardation ;	Developmental
	Myelofibrosis, idiopathic, ;Polycythemia vera, ;Thrombocythemia, essential, ;	Hematological
	Mycobacterial infection, atypical, familial disseminated, ;	Immunological
	STAT deficiency, complete ;	Unclassified
	Rubinstein-Taybi syndrome, ;	multiple
C16	Accelerated tumor formation, susceptibility to ;Breast cancer ;Colorectal cancer, ;Leukemia, Philadelphia chromosome-positive, resistant to imatinib ;Squamous cell carcinoma, head and neck, ;	Cancer
	Rubinstein-Taybi syndrome, ;	multiple
C17	Adenocarcinoma of lung, response to tyrosine kinase inhibitor in, ;Colorectal cancer ;Hepatoblastoma ;Hepatocellular carcinoma, ;Nonsmall cell lung cancer, response to tyrosine kinase inhibitor in, ;Ovarian carcinoma, endometrioid type ;Pilomatricoma, ;Squamous cell carcinoma, head and neck, ;	Cancer
	Loeys-Dietz syndrome, ;	Connective tissue
	Rubinstein-Taybi syndrome, ;	multiple
C18	Colorectal cancer ;Hepatoblastoma ;Hepatocellular carcinoma, ;Ovarian carcinoma, endometrioid type ;Pilomatricoma, ;Squamous cell carcinoma, head and neck, ;	Cancer
	Loeys-Dietz syndrome, ;	Connective tissue
	Cirrhosis, cryptogenic ;Cirrhosis, noncryptogenic, susceptibility to, ;	Gastrointestinal
	Rubinstein-Taybi syndrome, ;	multiple

C19	Accelerated tumor formation, susceptibility to ;Adenocarcinoma of lung, somatic, ;Bladder cancer, ;Burkitt lymphoma, ;Colorectal cancer, somatic ;Leukemia/lymphoma, B-cell, ;Melanoma, melignant, somatic ;Nonsmall cell lung cancer, somatic ;Retinoblastoma ;	Cancer
	Leprechaunism, ;	Developmental
	Diabetes mellitus, insulin-resistant, with acanthosis nigricans ;	Endocrine
	Cirrhosis, cryptogenic ;Cirrhosis, noncryptogenic, susceptibility to, ;	Gastrointestinal
	Rabson-Mendenhall syndrome, ;Rubinstein-Taybi syndrome, ;	multiple
C20	Adenocarcinoma of lung, response to tyrosine kinase inhibitor in, ;Bladder cancer, ;Burkitt lymphoma, ;Colorectal cancer ;Hepatoblastoma ;Hepatocellular carcinoma, ;Nonsmall cell lung cancer, response to tyrosine kinase inhibitor in, ;Ovarian carcinoma, endometrioid type ;Pilomatricoma, ;Retinoblastoma ;Squamous cell carcinoma, head and neck, ;	Cancer
	Loeys-Dietz syndrome, ;	Connective tissue
	Rubinstein-Taybi syndrome, ;	multiple
C21	Accelerated tumor formation, susceptibility to ;Adrenal adenoma, sporadic ;Bladder cancer, ;Breast cancer ;Burkitt lymphoma, ;Carcinoid tumor of lung ;Colorectal cancer ;Hepatoblastoma ;Hepatocellular carcinoma, ;Leukemia, Philadelphia chromosome-positive, resistant to imatinib ;Lipoma, sporadic ;Multiple endocrine neoplasia I ;Ovarian carcinoma, endometrioid type ;Parathyroid adenoma, sporadic ;Pilomatricoma, ;Retinoblastoma ;Squamous cell carcinoma, head and neck, ;	Cancer
	Loeys-Dietz syndrome, ;	Connective tissue
	Angiofibroma, sporadic ;Hyperparathyroidism, AD, ;Prolactinoma, hyperparathyroidism, carcinoid syndrome ;	Endocrine
	Rubinstein-Taybi syndrome, ;	multiple
C22	Adenocarcinoma of lung, response to tyrosine kinase inhibitor in, ;Colorectal cancer ;Hepatoblastoma ;Hepatocellular carcinoma, ;Nonsmall cell lung cancer, response to tyrosine kinase inhibitor in, ;Ovarian carcinoma, endometrioid type ;Pilomatricoma, ;Squamous cell carcinoma, head and neck, ;	Cancer
	Loeys-Dietz syndrome, ;	Connective tissue
	Rubinstein-Taybi syndrome, ;	multiple
C23	Bladder cancer, ;Breast cancer, sporadic ;Colorectal cancer ;Hepatoblastoma ;Hepatocellular carcinoma, ;Ovarian carcinoma, endometrioid type ;Pilomatricoma, ;Retinoblastoma ;Squamous cell carcinoma, head and neck, ;	Cancer
	Loeys-Dietz syndrome, ;	Connective tissue
	Rubinstein-Taybi syndrome, ;	multiple

C24	Accelerated tumor formation, susceptibility to ;Bladder cancer, ;Burkitt lymphoma, ;Colorectal cancer ;Hepatoblastoma ;Hepatocellular carcinoma, ;Leukemia/lymphoma, B-cell, ;Ovarian carcinoma, endometrioid type ;Pilomatricoma, ;Retinoblastoma ;	Cancer
	Leprechaunism, ;	Developmental
	Diabetes mellitus, insulin-resistant, with acanthosis nigricans ;	Endocrine
	Amyloidosis, Finnish type, ;	Neurological
	Rabson-Mendenhall syndrome, ;Rubinstein-Taybi syndrome, ;	multiple
C25	Colorectal cancer, ;Colorectal cancer ;Hepatoblastoma ;Hepatocellular carcinoma, ;Oligodontia-colorectal cancer syndrome, ;Ovarian carcinoma, endometrioid type ;Pilomatricoma, ;	Cancer
	Loeys-Dietz syndrome, ;	Connective tissue
	Rubinstein-Taybi syndrome, ;	multiple
C26	Adenocarcinoma of lung, response to tyrosine kinase inhibitor in, ;Colorectal cancer ;Hepatoblastoma ;Hepatocellular carcinoma, ;Nonsmall cell lung cancer, response to tyrosine kinase inhibitor in, ;Ovarian carcinoma, endometrioid type ;Pilomatricoma, ;Squamous cell carcinoma, head and neck, ;	Cancer
	Loeys-Dietz syndrome, ;	Connective tissue
	Intrauterine and postnatal growth retardation ;	Developmental
C27	Bladder cancer, ;Breast cancer, sporadic ;Colorectal cancer ;Hepatoblastoma ;Hepatocellular carcinoma, ;Ovarian carcinoma, endometrioid type ;Pilomatricoma, ;Retinoblastoma ;Squamous cell carcinoma, head and neck, ;	Cancer
	Loeys-Dietz syndrome, ;	Connective tissue
	Mycobacterial infection, atypical, familial disseminated, ;	Immunological
	STAT deficiency, complete ;	Unclassified
	Rubinstein-Taybi syndrome, ;	multiple
C28	Accelerated tumor formation, susceptibility to ;Colorectal cancer, ;Leukemia, Philadelphia chromosome-positive, resistant to imatinib ;Squamous cell carcinoma, head and neck, ;Wilms tumor, type , ;	Cancer
	Denys-Drash syndrome, ;Mesangial sclerosis, isolated diffuse, ;	Renal
	Frasier syndrome, ;Rubinstein-Taybi syndrome, ;WAGR syndrome, ;	multiple
C29	Colorectal cancer, ;Squamous cell carcinoma, head and neck, ;	Cancer
	Rubinstein-Taybi syndrome, ;	multiple

C30	Adenocarcinoma of lung, response to tyrosine kinase inhibitor in, ;Colorectal cancer ;Hemangioma, capillary infantile, somatic, ;Hepatoblastoma ;Hepatocellular carcinoma, ;Hepatocellular carcinoma, childhood type, ;Nonsmall cell lung cancer, response to tyrosine kinase inhibitor in, ;Ovarian carcinoma, endometrioid type ;Pilomatricoma, ;Renal cell carcinoma, papillary, familial and sporadic, ;	Cancer
	Loeys-Dietz syndrome, ;	Connective tissue
	Polycystic kidney disease, adult type I, ;	Renal
	Rubinstein-Taybi syndrome, ;	multiple
C31	Colorectal cancer ;Hepatoblastoma ;Hepatocellular carcinoma, ;Ovarian carcinoma, endometrioid type ;Pilomatricoma, ;	Cancer
	Intrauterine and postnatal growth retardation ;	Developmental
	Diabetes mellitus, noninsulin-dependent ;	Endocrine
	Cirrhosis, cryptogenic ;Cirrhosis, noncryptogenic, susceptibility to, ;	Gastrointestinal
	Mycobacterial infection, atypical, familial disseminated, ;	Immunological
	STAT deficiency, complete ;	Unclassified
	Rubinstein-Taybi syndrome, ;	multiple
C32	Colorectal cancer ;Hepatoblastoma ;Hepatocellular carcinoma, ;Ovarian carcinoma, endometrioid type ;Pilomatricoma, ;	Cancer
	Loeys-Dietz syndrome, ;	Connective tissue
C33	Colorectal cancer ;Hepatoblastoma ;Hepatocellular carcinoma, ;Ovarian carcinoma, endometrioid type ;Pilomatricoma, ;Squamous cell carcinoma, head and neck, ;	Cancer
	Loeys-Dietz syndrome, ;	Connective tissue
	Rubinstein-Taybi syndrome, ;	multiple
C34	Adenocarcinoma of lung, response to tyrosine kinase inhibitor in, ;Colorectal cancer ;Hepatoblastoma ;Hepatocellular carcinoma, ;Leukemia, Philadelphia chromosome-positive, resistant to imatinib ;Nonsmall cell lung cancer, response to tyrosine kinase inhibitor in, ;Ovarian carcinoma, endometrioid type ;Pilomatricoma, ;	Cancer
	Loeys-Dietz syndrome, ;	Connective tissue
	Leprechaunism, ;	Developmental
	Diabetes mellitus, insulin-resistant, with acanthosis nigricans ;	Endocrine
	Alexander disease, ;Rabson-Mendenhall syndrome, ;	multiple
C35	Adenocarcinoma of lung, response to tyrosine kinase inhibitor in, ;Colorectal cancer ;Hepatoblastoma ;Hepatocellular carcinoma, ;Nonsmall cell lung cancer, response to tyrosine kinase inhibitor in, ;Ovarian carcinoma, endometrioid type ;Pilomatricoma, ;Squamous cell carcinoma, head and neck, ;	Cancer
	Loeys-Dietz syndrome, ;	Connective tissue
	Rubinstein-Taybi syndrome, ;	multiple

C36	Accelerated tumor formation, susceptibility to ;Adenocarcinoma of lung, response to tyrosine kinase inhibitor in, ;Bladder cancer, ;Burkitt lymphoma, ;Colorectal cancer ;Hepatoblastoma ;Hepatocellular carcinoma, ;Leukemia, acute myeloblastic ;Nonsmall cell lung cancer, response to tyrosine kinase inhibitor in, ;Ovarian carcinoma, endometrioid type ;Pilomatricoma, ;Retinoblastoma ;	Cancer
	Loeys-Dietz syndrome, ;	Connective tissue
	Intrauterine and postnatal growth retardation ;	Developmental
	Myelofibrosis, idiopathic, ;Polycythemia vera, ;Thrombocythemia, essential, ;	Hematological
	Mycobacterial infection, atypical, familial disseminated, ;Neutrophil immunodeficiency syndrome, ;	Immunological
	STAT deficiency, complete ;	Unclassified
	Rubinstein-Taybi syndrome, ;	multiple
C37	Colorectal cancer ;Hepatoblastoma ;Hepatocellular carcinoma, ;Ovarian carcinoma, endometrioid type ;Pilomatricoma, ;Squamous cell carcinoma, head and neck, ;	Cancer
	Loeys-Dietz syndrome, ;	Connective tissue
C38	Accelerated tumor formation, susceptibility to ;Breast cancer ;Breast cancer, sporadic ;Breast cancer, susceptibility to, ;Colorectal cancer, ;Leukemia, acute myeloid, ;Leukemia, acute promyelocytic, NPM/RARA type ;Leukemia/lymphoma, B-cell, ;Leukemia, Philadelphia chromosome-positive, resistant to imatinib ;Squamous cell carcinoma, head and neck, ;Wilms tumor, type , ;	Cancer
	Rheumatoid arthritis, systemic juvenile, susceptibility to, ;	Connective tissue
	DNA topoisomerase I, camptothecin-resistant ;	Metabolic
	Spinocerebellar ataxia , ;	Neurological
	Denys-Drash syndrome, ;Mesangial sclerosis, isolated diffuse, ;	Renal
	Frasier syndrome, ;Prader-Willi syndrome, ;Prader-Willi syndrome, ;Rubinstein-Taybi syndrome, ;WAGR syndrome, ;	multiple
C39	Adenocarcinoma of lung, response to tyrosine kinase inhibitor in, ;Burkitt lymphoma, ;Colorectal cancer ;Hepatoblastoma ;Hepatocellular carcinoma, ;Nonsmall cell lung cancer, response to tyrosine kinase inhibitor in, ;Ovarian carcinoma, endometrioid type ;Pilomatricoma, ;Squamous cell carcinoma, head and neck, ;	Cancer
	Loeys-Dietz syndrome, ;	Connective tissue
	Rubinstein-Taybi syndrome, ;	multiple
C40	Accelerated tumor formation, susceptibility to ;Burkitt lymphoma, ;Colorectal cancer, ;Leukemia/lymphoma, B-cell, ;	Cancer
	Leprechaunism, ;	Developmental
	Diabetes mellitus, insulin-resistant, with acanthosis nigricans ;	Endocrine
	Rabson-Mendenhall syndrome, ;Rubinstein-Taybi syndrome, ;	multiple

C41	Adenocarcinoma of lung, response to tyrosine kinase inhibitor in, ;Colorectal cancer ;Hepatoblastoma ;Hepatocellular carcinoma, ;Nonsmall cell lung cancer, response to tyrosine kinase inhibitor in, ;Ovarian carcinoma, endometrioid type ;Pilomatricoma, ;Squamous cell carcinoma, head and neck, ;	Cancer
	Loeys-Dietz syndrome, ;	Connective tissue
	Rubinstein-Taybi syndrome, ;	multiple
C42	Colorectal cancer ;Hepatoblastoma ;Hepatocellular carcinoma, ;Ovarian carcinoma, endometrioid type ;Pilomatricoma, ;	Cancer
	Loeys-Dietz syndrome, ;	Connective tissue
	Rubinstein-Taybi syndrome, ;	multiple
C43	Burkitt lymphoma, ;Colorectal cancer ;Hepatoblastoma ;Hepatocellular carcinoma, ;Ovarian carcinoma, endometrioid type ;Pilomatricoma, ;Squamous cell carcinoma, head and neck, ;	Cancer
	Loeys-Dietz syndrome, ;	Connective tissue
	Rubinstein-Taybi syndrome, ;	multiple
C44	Accelerated tumor formation, susceptibility to ;Breast cancer ;Breast cancer, sporadic ;Breast cancer, susceptibility to, ;Colorectal cancer, ;Leukemia, acute myeloid, ;Leukemia, acute promyelocytic, NPM/RARA type ;Leukemia/lymphoma, B-cell, ;Leukemia, Philadelphia chromosome-positive, resistant to imatinib ;Squamous cell carcinoma, head and neck, ;Wilms tumor, type , ;	Cancer
	Rheumatoid arthritis, systemic juvenile, susceptibility to, ;	Connective tissue
	DNA topoisomerase I, camptothecin-resistant ;	Metabolic
	Spinocerebellar ataxia , ;	Neurological
	Denys-Drash syndrome, ;Mesangial sclerosis, isolated diffuse, ;	Renal
	Frasier syndrome, ;Prader-Willi syndrome, ;Prader-Willi syndrome, ;Rubinstein-Taybi syndrome, ;WAGR syndrome, ;	multiple
C45	Accelerated tumor formation, susceptibility to ;Colorectal cancer, ;Leukemia/lymphoma, B-cell, ;	Cancer
	Leprechaunism, ;	Developmental
	Diabetes mellitus, insulin-resistant, with acanthosis nigricans ;	Endocrine
	Rabson-Mendenhall syndrome, ;Rubinstein-Taybi syndrome, ;	multiple
C46	Colorectal cancer ;Hepatoblastoma ;Hepatocellular carcinoma, ;Ovarian carcinoma, endometrioid type ;Pilomatricoma, ;Squamous cell carcinoma, head and neck, ;	Cancer
	Rubinstein-Taybi syndrome, ;	multiple

C47	Accelerated tumor formation, susceptibility to ;Adenocarcinoma of lung, response to tyrosine kinase inhibitor in, ;Adenocarcinoma of lung, somatic, ;Bladder cancer, ;Burkitt lymphoma, ;Colorectal cancer ;Colorectal cancer, somatic ;Hepatoblastoma ;Hepatocellular carcinoma, ;Leukemia, acute myeloblastic ;Melanoma, melignant, somatic ;Nonsmall cell lung cancer, response to tyrosine kinase inhibitor in, ;Nonsmall cell lung cancer, somatic ;Ovarian carcinoma, endometrioid type ;Pilomatricoma, ;Retinoblastoma ;	Cancer
	Epidermolysis bullosa, generalized atrophic benign, ;Epidermolysis bullosa, junctional, with pyloric atresia, ;Epidermolysis bullosa, junctional, with pyloric stenosis, ;Epidermolysis bullosa of hands and feet, ;	Dermatological
	Intrauterine and postnatal growth retardation ;	Developmental
	Diabetes mellitus, noninsulin-dependent ;	Endocrine
	Myelofibrosis, idiopathic, ;Polycythemia vera, ;Thrombocythemia, essential, ;	Hematological
	Mycobacterial infection, atypical, familial disseminated, ;	Immunological
	Obesity, susceptibility to, ;	Nutritional
	Asthma, nocturnal, susceptibility to ;	Respiratory
	Beta--adrenoreceptor agonist, reduced response to ;STAT deficiency, complete ;	Unclassified
	Rubinstein-Taybi syndrome, ;	multiple
C48	Accelerated tumor formation, susceptibility to ;Adrenal adenoma, sporadic ;Breast cancer ;Burkitt lymphoma, ;Carcinoid tumor of lung ;Colorectal cancer ;Hepatoblastoma ;Hepatocellular carcinoma, ;Leukemia, Philadelphia chromosome-positive, resistant to imatinib ;Lipoma, sporadic ;Multiple endocrine neoplasia I ;Ovarian carcinoma, endometrioid type ;Parathyroid adenoma, sporadic ;Pilomatricoma, ;Squamous cell carcinoma, head and neck, ;	Cancer
	Loeys-Dietz syndrome, ;	Connective tissue
	Angiofibroma, sporadic ;Hyperparathyroidism, AD, ;Prolactinoma, hyperparathyroidism, carcinoid syndrome ;	Endocrine
	Rubinstein-Taybi syndrome, ;	multiple
C49	Adenocarcinoma of lung, response to tyrosine kinase inhibitor in, ;Colorectal cancer, ;Nonsmall cell lung cancer, response to tyrosine kinase inhibitor in, ;Squamous cell carcinoma, head and neck, ;	Cancer
	Rubinstein-Taybi syndrome, ;	multiple
C50	Adenocarcinoma of lung, response to tyrosine kinase inhibitor in, ;Colorectal cancer, ;Leukemia, Philadelphia chromosome-positive, resistant to imatinib ;Nonsmall cell lung cancer, response to tyrosine kinase inhibitor in, ;Squamous cell carcinoma, head and neck, ;	Cancer
	Intrauterine and postnatal growth retardation ;	Developmental

	Rubinstein-Taybi syndrome, ;	multiple
C51	Accelerated tumor formation, susceptibility to ;Burkitt lymphoma, ;Colorectal cancer, ;Leukemia/lymphoma, B-cell, ;	Cancer
	Leprechaunism, ;	Developmental
	Diabetes mellitus, insulin-resistant, with acanthosis nigricans ;	Endocrine
	Rabson-Mendenhall syndrome, ;Rubinstein-Taybi syndrome, ;	multiple
C52	Accelerated tumor formation, susceptibility to ;Adenocarcinoma of lung, somatic, ;Bladder cancer, ;Colorectal cancer, somatic ;Leukemia/lymphoma, B-cell, ;Melanoma, melignant, somatic ;Nonsmall cell lung cancer, somatic ;Retinoblastoma ;	Cancer
	Leprechaunism, ;	Developmental
	Diabetes mellitus, insulin-resistant, with acanthosis nigricans ;Hyperproinsulinemia, familial ;MODY, one form, ;	Endocrine
	Cirrhosis, cryptogenic ;Cirrhosis, noncryptogenic, susceptibility to, ;	Gastrointestinal
	Rabson-Mendenhall syndrome, ;Rubinstein-Taybi syndrome, ;	multiple
C53	Colorectal cancer ;Hepatoblastoma ;Hepatocellular carcinoma, ;Ovarian carcinoma, endometrioid type ;Pilomatricoma, ;Squamous cell carcinoma, head and neck, ;	Cancer
	Loeys-Dietz syndrome, ;	Connective tissue
	Rubinstein-Taybi syndrome, ;	multiple
C54	Accelerated tumor formation, susceptibility to ;Bladder cancer, ;Burkitt lymphoma, ;Colorectal cancer ;Hepatoblastoma ;Hepatocellular carcinoma, ;Leukemia/lymphoma, B-cell, ;Ovarian carcinoma, endometrioid type ;Pilomatricoma, ;Retinoblastoma ;	Cancer
	Leprechaunism, ;	Developmental
	Diabetes mellitus, insulin-resistant, with acanthosis nigricans ;	Endocrine
	Amyloidosis, Finnish type, ;	Neurological
	Rabson-Mendenhall syndrome, ;Rubinstein-Taybi syndrome, ;	multiple
C55	Adenocarcinoma of lung, somatic, ;Colorectal cancer, somatic ;Melanoma, melignant, somatic ;Nonsmall cell lung cancer, somatic ;	Cancer
	Diabetes mellitus, noninsulin-dependent ;	Endocrine
	Cirrhosis, cryptogenic ;Cirrhosis, noncryptogenic, susceptibility to, ;	Gastrointestinal
	Rubinstein-Taybi syndrome, ;	multiple
C56	Adenocarcinoma of lung, response to tyrosine kinase inhibitor in, ;Colorectal cancer ;Hepatoblastoma ;Hepatocellular carcinoma, ;Nonsmall cell lung cancer, response to tyrosine kinase inhibitor in, ;Ovarian carcinoma, endometrioid type ;Pilomatricoma, ;Squamous cell carcinoma, head and neck, ;	Cancer
	Rubinstein-Taybi syndrome, ;	multiple

C57	Burkitt lymphoma, ;Colorectal cancer ;Hepatoblastoma ;Hepatocellular carcinoma, ;Ovarian carcinoma, endometrioid type ;Pilomatricoma, ;Squamous cell carcinoma, head and neck, ;	Cancer
	Loeys-Dietz syndrome, ;	Connective tissue
	Rubinstein-Taybi syndrome, ;	multiple
C58	Accelerated tumor formation, susceptibility to ;Adrenal adenoma, sporadic ;Bladder cancer, ;Breast cancer ;Burkitt lymphoma, ;Carcinoid tumor of lung ;Colorectal cancer ;Hepatoblastoma ;Hepatocellular carcinoma, ;Leukemia/lymphoma, B-cell, ;Leukemia, Philadelphia chromosome-positive, resistant to imatinib ;Lipoma, sporadic ;Multiple endocrine neoplasia I ;Ovarian carcinoma, endometrioid type ;Parathyroid adenoma, sporadic ;Pilomatricoma, ;Retinoblastoma ;Squamous cell carcinoma, head and neck, ;	Cancer
	Loeys-Dietz syndrome, ;	Connective tissue
	Angiofibroma, sporadic ;Hyperparathyroidism, AD, ;Prolactinoma, hyperparathyroidism, carcinoid syndrome ;	Endocrine
	Cirrhosis, cryptogenic ;Cirrhosis, noncryptogenic, susceptibility to, ;	Gastrointestinal
	Rubinstein-Taybi syndrome, ;	multiple
C59	Adenocarcinoma of lung, response to tyrosine kinase inhibitor in, ;Colorectal cancer, ;Nonsmall cell lung cancer, response to tyrosine kinase inhibitor in, ;	Cancer
	Cirrhosis, cryptogenic ;Cirrhosis, noncryptogenic, susceptibility to, ;	Gastrointestinal
	Rubinstein-Taybi syndrome, ;	multiple
C60	Rickets, vitamin D-resistant, type IIA, ;Rickets, vitamin D-resistant, type IIB, ;	Bone
	Adenocarcinoma of lung, response to tyrosine kinase inhibitor in, ;Burkitt lymphoma, ;Colorectal cancer ;Hepatoblastoma ;Hepatocellular carcinoma, ;Nonsmall cell lung cancer, response to tyrosine kinase inhibitor in, ;Ovarian carcinoma, endometrioid type ;Pilomatricoma, ;Squamous cell carcinoma, head and neck, ;	Cancer
	Intrauterine and postnatal growth retardation ;	Developmental
	Rubinstein-Taybi syndrome, ;	multiple
